# Digital health technology burden and frustration among patients with multimorbidity

**DOI:** 10.1093/jamia/ocag090

**Published:** 2026-06-23

**Authors:** Haoxin Chen, Jiancheng Ye

**Affiliations:** Weill Cornell Medicine, Cornell University, New York, NY, United States; The University of Hong Kong, Hong Kong, China; Weill Cornell Medicine, Cornell University, New York, NY, United States; Feinberg School of Medicine, Northwestern University, Chicago, IL, United States

**Keywords:** digital health technology, multimorbidity, treatment burden, patient experience, digital divide

## Abstract

**Objectives:**

This study aimed to characterize patterns of digital health technology (DHT) use and examine the relationships between multimorbidity, DHT adoption, and user-reported frustration, with a focus on identifying socioeconomic and phenotypic determinants of digital health burden.

**Materials and Methods:**

A cross-sectional analysis was conducted using nationally representative public data from Health Information National Trends Survey (HINTS) 7. Adults with at least one chronic condition were included (*N* = 3753). The outcomes were the number of DHTs used and frustration with digital tools. Multivariable logistic regression models were employed, adjusting for sociodemographic and clinical variables. Phenotypic subgroup analyses were conducted based on multimorbidity, DHT use, and frustration patterns.

**Results:**

Among 3753 participants, 46.9% had multimorbidity. Participants with multimorbidity reported using a greater number of DHTs on average (mean = 3.9 vs 3.6, *P* < .001) compared to those with a single condition, yet exhibited significantly higher rates of frustration with digital tasks (61.2% vs 54.1%, *P* < .001). Both higher income and educational groups were associated with lower odds of frustration. Greater DHT use was also independently associated with reduced frustration (OR = 0.78, 95% CI: 0.71-0.87, *P* < .01). Phenotypic subgroup analysis further identified individuals with multimorbidity and low DHT versatility as the most vulnerable profile, characterized by older age, lower socioeconomic status, and the highest frustration prevalence (60.3%).

**Conclusion:**

While individuals with multimorbidity use more DHTs, they experience greater frustration, particularly those with lower socioeconomic status. However, higher engagement with DHTs (as measured by number of technology types adopted) is associated with lower frustration, suggesting that technology proficiency may mitigate burden. Targeted, equity-conscious interventions, including competency-based digital literacy programs, simplified user-centered design, and structural supports addressing device access and connectivity, are needed to reduce digital health burden and improve outcomes for patients living with multiple chronic conditions.

## Introduction

Digital health technologies (DHTs), including patient portals, telehealth platforms, mobile health applications, and wearable devices, are becoming deeply embedded in the infrastructure of chronic disease management. While these tools have the potential to expand access, support self-management, and enable remote monitoring, their real-world impact on patient workload, experience, and outcomes remains inconsistent and complex.^1^ This complexity is particularly evident among the growing population living with multimorbidity—defined as having 2 or more chronic conditions.^2^ These individuals navigate inherently complex care regimens, experience fragmented care delivery, and shoulder substantial self-management responsibilities.^3^ The layering of multiple, often condition-specific DHTs onto these existing demands creates a critical juncture that may either streamline care coordination and empower patients, or unintentionally amplify their treatment burden.^4^ Understanding the determinants of these divergent pathways, where DHTs facilitate vs hinder care, is, therefore, important to achieving patient-centered implementation.^5^

Emerging evidence demonstrates that DHTs can improve selected clinical outcomes and care processes in specific chronic conditions.^6^ However, these benefits are not universal. Effects are heterogeneous and contingent on factors such as usability, integration into clinical workflows, and surrounding support models.^7^^,^^8^ Reviews of digital interventions and implementation efforts in chronic disease management highlight benefits for patient engagement and provider coordination, yet also underscore barriers such as limited digital literacy, poor workflow fit, and fragmented digital ecosystems that shift administrative work from clinics to patients and families.^9^ Studies document patient frustration with non-interoperable systems requiring duplicate data entry across multiple portals and the cognitive burden of synthesizing fragmented health information.^10^ Despite this growing recognition of digital barriers, a substantial gap persists. Few studies have systematically quantified patient-reported frustration with DHTs or identified specific vulnerability phenotypes using large, nationally representative datasets. This leaves an insufficient understanding of how these digital barriers manifest and are distributed across the population, particularly among patients with complex chronic care needs.^11^ The Burden of Treatment (BoT) framework offers a valuable lens through which to examine this issue.^12^ It conceptualizes health-related work as a function of the workload imposed by healthcare (eg, learning new technologies, monitoring symptoms, scheduling virtual appointments) and the capacity individuals have to manage it (eg, skills, resources, social support).^9^ For individuals with multimorbidity, who often juggle multiple portals, devices, and applications, the workload imposed by DHTs can be substantial, especially when technologies are numerous, functionally overlapping, poorly integrated, or misaligned with their needs and capacities.^13^ This raises a pressing concern that the rapid expansion of DHTs, if not designed and implemented with equity as a core principle, risks widening existing health disparities by failing to meet the needs of the most vulnerable groups. A recent scoping review focused on multimorbidity has called for more standardized approaches to characterize the types and intensity of DHT use, document patients’ negative experiences, and evaluate the overall balance of benefits vs burdens.^11^

To address these gaps, this study goes beyond characterizing DHT adoption rates to systematically quantify patient-reported frustration—a critical but understudied barrier to digital health equity. While prior HINTS-based research has documented socioeconomic gradients in portal use and digital literacy, few studies have examined frustration as a distinct outcome or explored how the interplay between clinical complexity, technology proficiency, and socioeconomic factors shapes digital burden. Our phenotyping approach provides a framework for identifying specific vulnerable subgroups (eg, older adults with multimorbidity and low DHT versatility) who face compounded digital barriers, moving beyond descriptive disparities to actionable patient profiles for targeted intervention design. This paradigm is widely employed in epidemiological studies, such as those utilizing the National Health and Nutrition Examination Survey (NHANES) to define the prevalence and subclassifications of metabolic dysfunction-associated steatotic liver disease under new consensus nomenclature,^14^ and to investigate the relationship between serum biomarkers and obesity phenotypes using multivariable logistic regression and restricted cubic splines.^15^ Similarly, the use of large-scale longitudinal cohorts such as the China Health and Retirement Longitudinal Study (CHARLS) to examine the association between sarcopenia and frailty through multivariable regression and subgroup analysis demonstrates the robustness of such methods for identifying at-risk subgroups in aging populations.^16^

Building upon these foundations, this study aims to comprehensively assess DHT usage and burden among adults with multimorbidity using a nationally representative dataset. We first quantify the number and types of DHTs used by this population, then model the association between multimorbidity status and DHT use, while simultaneously examining the association with patient-reported frustration. Furthermore, we conduct phenotypic analyses through stratification to identify vulnerable subgroups. This research seeks to generate evidence that can inform pragmatic, equity-minded strategies to optimize digital care for people with multiple chronic conditions, maximizing the benefits of technology while minimizing associated workload. In this study, we use 3 related but distinct terms with precision. Technology adoption refers to the decision to incorporate one or more DHTs into one’s care routines—capturing whether a given technology has been taken up at all. Technology use refers to the active application of DHTs for health purposes, operationalized here as the total count of distinct technology types employed. Digital engagement refers to the quality, depth, and regularity of a person’s ongoing interactions with DHTs, encompassing both adoption and use as well as the subjective experience of that interaction, including user-reported confidence and frustration.^9^^,^^11^

## Methods

### Study design

We conducted a cross-sectional analysis of data from the Health Information National Trends Survey (HINTS 7)^17^ from the National Cancer Institute. HINTS surveys are administered biennially among noninstitutionalized US civilians aged ≥18 years and contain nationally representative data on awareness and use of health-related information. The HINTS 7 sample comprised 7278 respondents, with surveys conducted from March 25, 2024, to September 16, 2024. We excluded observations with missing, erroneous, or inapplicable values, resulting in a final analytical sample of 3753 participants.

### Measures

Multimorbidity was operationalized based on a count of self-reported chronic conditions, including diabetes, hypertension, heart disease, and lung disease. Participants reporting zero chronic conditions were excluded from subsequent analyses. DHT use was assessed through survey items covering several domains: patient portal functions (eg, information lookup, secure messaging, accessing test results, and appointment scheduling), health and wellness applications, organizer or portal apps, wearable devices, and sharing of device-generated data with healthcare providers. Responses were summed to create a continuous variable ranging from 0 to 8, representing the total number of distinct technology types used. Frustration with DHTs was measured by recoding agreement with the statement that digital tasks are frustrating into a binary variable, where 1 indicated “somewhat agree” or “strongly agree” and 0 indicated “somewhat disagree” or “strongly disagree.”

Key sociodemographic covariates were included: age, sex, race/ethnicity, education, household income, and health insurance status. Age was modeled continuously in regression analyses to maximize statistical power and detect linear trends. Education and income were recategorized into 3 ordinal levels to enhance interpretability while maintaining sufficient cell sizes. Education was grouped as: (1) high school or less, (2) some college or associate degree, and (3) bachelor’s degree or higher. Income was categorized as: (1) $0–$19 999, (2) $20 000–$74 999, and (3) ≥$75 000. All categorical variables were dummy coded with explicit reference categories specified in the regression models.

### Descriptive analysis

Descriptive analyses were conducted to summarize the prevalence of multimorbidity, the distribution of DHT use, and participant demographics. Continuous variables are presented as mean ± standard deviation (SD) and compared using independent samples t-tests. Categorical variables are reported as frequencies and percentages [*n* (%)] and compared using chi-square tests. Statistical significance was defined as *P* < .05 for all analyses.

### Regression models

Two multivariable logistic regression models were employed. Model 1 assessed the association between DHT use, frustration with DHTs, and multimorbidity status, adjusted for sociodemographic factors. Specifically, a logistic regression model was fitted with multimorbidity as the outcome, regressed on age, income, education, health insurance status, sex, race/ethnicity, the number of DHTs used, and frustration with DHTs. This model estimated adjusted odds ratios (aORs) with 95% confidence intervals (CIs) for the factors associated with having multimorbidity.


Multimorbidity ∼ Age + Income + Education + Insurance + Sex + Race/Ethnicity + Number of DHTs + Frustration   (Model 1)

Model 2 evaluated the association between multimorbidity status, DHT use, and frustration with digital tasks, controlling for the same set of covariates. Using logistic regression, frustration (binary: 0 = not frustrated, 1 = frustrated) was the outcome, with multimorbidity status, the number of DHTs used, and all sociodemographic variables included as independent variables. The aORs with corresponding 95% CIs were computed to quantify these relationships.


Frustration ∼ Multimorbidity + Number of DHTs + Age + Income + Education + Insurance + Sex + Race/Ethnicity   (Model 2)

For both models, multicollinearity was assessed using variance inflation factors (VIF), and model fit was evaluated using the Hosmer-Lemeshow goodness-of-fit test.

### Subgroup analysis

To further explore the interplay between multimorbidity, DHT use, and user frustration, we conducted subgroup analyses based on 2 distinct 2 × 2 grouping strategies to identify clinically relevant phenotypes.

First grouping strategy: Participants were classified by multimorbidity status (single vs multiple conditions) and level of DHT adoption. We used a cutoff of 6 technologies (75th percentile) to define higher versatility (≥6 DHTs) vs lower versatility (<6 DHTs). This threshold was selected based on 3 considerations: (1) distribution properties, which provided adequate sample sizes across all 4 groups while capturing meaningful variation in adoption intensity; (2) interpretability, which represented engagement with multiple technology types beyond basic portal functions; and (3) sensitivity preliminary analyses using alternative cutoffs (median split at 4 technologies, 90th percentile at 7 technologies) yielded similar frustration patterns and phenotype characteristics (results available upon request). While this threshold is data-driven rather than theoretically predetermined, the consistency of findings across cutpoints suggests robustness. Differences in frustration levels and other key characteristics across the resulting 4 groups were assessed using one-way ANOVA for continuous variables (with post hoc Tukey tests where appropriate) or Kruskal-Wallis tests for non-normally distributed data, and chi-square tests for categorical variables.

Second grouping strategy: Participants were categorized by multimorbidity status (single vs multiple conditions) and the presence or absence of frustration (binary: not frustrated vs frustrated). For this stratification, the outcome was the level of DHT use. Group differences in the number of technologies utilized, along with other sociodemographic variables, were evaluated using one-way ANOVA or Kruskal-Wallis tests as appropriate.

For both grouping approaches, we profiled the resulting subgroups by examining the distribution of sociodemographic characteristics. Additionally, to visually represent patterns of specific DHT usage frequencies across the 4 groups within each classification, radar plots were generated using normalized scores, providing a comparative overview of technology engagement behaviors across multiple dimensions of digital health literacy and operational competence.

### Study approval

This study was deemed exempt from institutional review board approval as it utilized only publicly available, de-identified data from the HINTS.

## Results

### Descriptive analysis

Descriptive statistics for the study participants, stratified by multimorbidity status, are presented in Table 1. The analytical sample comprised 1990 individuals with a single chronic condition and 1763 individuals with multimorbidity (2 or more conditions), representing 53.1% and 46.9% of the sample, respectively.

**Table 1.** Characteristics of study participants stratified by multimorbidity status.

Variables	Total	One condition	Two or more conditions	*P*-value
	*N* = 3753	*n* = 1990	*n* = 1763	
Age, years, mean (SD)	60.0 ± 20.2	57.8 ± 20.6	62.5 ± 19.6	**<.001**
Number of conditions, mean (SD)	1.6 ± 0.8	1.0 ± 0.0	2.3 ± 0.6	**<.001**
Types of technology used, mean (SD)	3.7 ± 2.2	3.9 ± 2.1	3.6 ± 2.3	**<.001**
Income ranges, *N* (%)				**<.001**
$0–$19 999	720 (21.0)	315 (17.2)	405 (25.3)	
$20 000–$74 999	1509 (44.0)	767 (42.0)	742 (46.3)	
≥$75 000	1199 (35.0)	745 (40.8)	454 (28.4)	
Education, *N* (%)				**<.001**
High school or lower	951 (26.2)	445 (23.1)	506 (29.7)	
High school graduate/some college	1180 (32.5)	592 (30.7)	588 (34.6)	
Bachelor or higher	1498 (41.3)	890 (46.2)	608 (35.7)	
Race, *N* (%)				**<.001**
Hispanic	606 (17.6)	336 (18.3)	270 (16.8)	
Asian	142 (4.1)	84 (4.6)	58 (3.6)	
Black	656 (19.1)	307 (16.7)	349 (21.7)	
White	1920 (55.8)	1058 (57.5)	862 (53.7)	
Other	120 (3.5)	54 (2.9)	66 (4.1)	
Sex, *N* (%)				.056
Female	2119 (58.3)	1153 (59.8)	966 (56.6)	
Male	1517 (41.7)	776 (40.2)	741 (43.4)	
Health insurance, *N* (%)	3494 (93.1)	1834 (92.2)	1660 (94.2)	**.038**
Diabetes, *N* (%)	1521 (40.5)	284 (14.3)	1237 (70.2)	**<.001**
High blood pressure, *N* (%)	2953 (78.7)	1322 (66.4)	1631 (92.5)	**<.001**
Heart condition, *N* (%)	720 (19.2)	86 (4.3)	634 (36.0)	**<.001**
Lung disease, *N* (%)	914 (24.4)	298 (15.0)	616 (35.0)	**<.001**

Bold values indicate statistical significance.

Significant differences were observed between the 2 groups across sociodemographic, healthcare access, and technology-related variables. Participants with multimorbidity were significantly older than those with a single condition (mean [SD] age: 62.5 ± 19.6 years vs 57.8 ± 20.6 years, *P* < .001). Racial composition differed significantly between groups (*P* < .001); the proportion of Black individuals was higher in the multimorbidity group compared to the single condition group (21.7% vs 16.7%), while the proportion of White individuals was correspondingly lower (53.7% vs 57.5%). Income distribution differed significantly, with a higher prevalence of high household income (≥$75 000) observed in the single condition group compared to the multimorbidity group (40.8% vs 28.4%, *P* < .001). Education level also varied markedly, as participants with a single condition exhibited a lower proportion of individuals with high school education or less (23.1% vs 29.7%, *P* < .001) and a correspondingly higher proportion with bachelor’s degrees or higher (46.2% vs 35.7%).

Regarding DHT use, individuals with a single chronic condition reported using fewer types of technologies compared to those with multimorbidity (mean = 3.6 ± 2.1 vs 3.9 ± 2.3, *P* < .001). Significant differences were also observed in health insurance coverage, with a slightly higher proportion of insured individuals in the multimorbidity group (94.2% vs 92.2%, *P* = .038). The prevalence of specific chronic conditions differed substantially between groups, with the multimorbidity group showing higher rates of diabetes (70.2% vs 14.3%, *P* < .001), high blood pressure (92.5% vs 66.4%, *P* < .001), heart conditions (36.0% vs 4.3%, *P* < .001), and lung disease (35.0% vs 15.0%, *P* < .001).

Table 2 presents detailed DHT usage patterns stratified by multimorbidity status. In terms of specific DHT activities, the single condition group demonstrated higher rates of using the internet for health information (83.7% vs 79.6%, *P* = .002), making appointments online (65.7% vs 59.1%, *P* < .001), using wellness apps (52.9% vs 48.8%, *P* = .016), and using wearable devices to track health (34.8% vs 29.7%, *P* < .001). Conversely, individuals with multimorbidity were more likely to share health device information with healthcare providers (32.2% vs 22.2%, *P* < .001). The single condition group reported higher daily social media usage (55.9% vs 48.8%, *P* < .001) and lower rates of never using social media (18.8% vs 24.8%). Similarly, they were more likely to watch health-related videos on social media (*P* < .001). Regarding technology-related frustration, the multimorbidity group had a higher proportion strongly agreeing with feeling frustrated (22.5% vs 16.4%, *P* < .001) and a lower percentage strongly disagreeing (16.0% vs 22.0%). Both groups demonstrated high utilization of administrative portal functions—messaging providers (≈65%) and viewing test results (≈74%), with no significant differences, suggesting these have become standard features of chronic disease management. However, the single condition group more frequently made appointments online (65.7% vs 59.1%, *P* < .001), potentially reflecting greater comfort with transactional digital tasks or differences in care complexity affecting scheduling needs. Notably, social media engagement for health purposes remained limited across both groups, with over 83% never sharing personal health information and 72% never sharing general health content. This low engagement persists despite historical encouragement of peer support communities, particularly for cancer patients, suggesting either privacy concerns, platform preferences shifting away from traditional social media, or disconnect between recommended and actual patient behaviors in digital health information exchange.

**Table 2.** Digital health technology usage patterns and social media engagement stratified by multimorbidity status.

Variables	Total	One condition	Two or more conditions	*P*-value
	*N* = 3753	*n* = 1990	*n* = 1763	
Used internet for health information, *N* (%)	2846 (81.8)	1574 (83.7)	1272 (79.6)	**.002**
Used internet to send message, *N* (%)	2275 (65.5)	1249 (66.6)	1026 (64.3)	.175
Used internet to view results, *N* (%)	2580 (74.4)	1404 (74.9)	1176 (73.9)	.522
Used internet to make appointment, *N* (%)	2175 (62.4)	1233 (65.7)	942 (59.1)	**<.001**
Used wellness app, *N* (%)	1808 (50.9)	1006 (52.9)	802 (48.8)	**.016**
Used portal organizer app, *N* (%)	195 (5.3)	96 (4.9)	99 (5.7)	.299
Used wearable device to track health, *N* (%)	1200 (32.3)	685 (34.8)	515 (29.7)	**<.001**
Shared health device information, *N* (%)	984 (27.2)	433 (22.2)	551 (32.2)	**<.001**
Visited social media, *N* (%)				**<.001**
Almost every day	1946 (52.5)	1098 (55.9)	848 (48.8)	
At least once a week	432 (11.7)	225 (11.5)	207 (11.9)	
A few times a month	286 (7.7)	156 (7.9)	130 (7.5)	
Less than once a month	239 (6.5)	117 (6.0)	122 (7.0)	
Never	801 (21.6)	369 (18.8)	432 (24.8)	
Shared personal health information on social media, *N* (%)				.789
Almost every day	58 (1.6)	28 (1.4)	30 (1.7)	
At least once a week	78 (2.1)	37 (1.9)	41 (2.4)	
A few times a month	112 (3.0)	58 (3.0)	54 (3.1)	
Less than once a month	342 (9.3)	183 (9.4)	159 (9.2)	
Never	3096 (84.0)	1651 (84.4)	1445 (83.6)	
Shared general health information on social media, *N* (%)				.098
Almost every day	55 (1.5)	25 (1.3)	30 (1.7)	
At least once a week	103 (2.8)	45 (2.3)	58 (3.4)	
A few times a month	221 (6.0)	116 (5.9)	105 (6.1)	
Less than once a month	642 (17.4)	362 (18.5)	280 (16.2)	
Never	2664 (72.3)	1408 (72.0)	1256 (72.6)	
Interacted with similar people on social media, *N* (%)				.107
Almost every day	57 (1.5)	29 (1.5)	28 (1.6)	
At least once a week	125 (3.4)	61 (3.1)	64 (3.7)	
A few times a month	204 (5.5)	96 (4.9)	108 (6.2)	
Less than once a month	452 (12.2)	260 (13.3)	192 (11.0)	
Never	2858 (77.3)	1511 (77.2)	1347 (77.5)	
Watched health video on social media, *N* (%)				**<.001**
Almost every day	144 (3.9)	75 (3.8)	69 (4.0)	
At least once a week	334 (9.0)	183 (9.3)	151 (8.7)	
A few times a month	642 (17.3)	357 (18.2)	285 (16.4)	
Less than once a month	1057 (28.6)	608 (31.0)	449 (25.8)	
Never	1524 (41.2)	737 (37.6)	787 (45.2)	
Felt frustrating using new technology, *N* (%)				**<.001**
Strongly agree	713 (19.3)	323 (16.4)	390 (22.5)	
Somewhat agree	1413 (38.2)	742 (37.7)	671 (38.7)	
Somewhat disagree	867 (23.4)	469 (23.8)	398 (22.9)	
Strongly disagree	710 (19.2)	433 (22.0)	277 (16.0)	

Bold values indicate statistical significance.

However, no significant differences were detected in several other DHT-related activities between the 2 groups. These included using the internet to send secure messages to providers, viewing test results online, using portal organizer apps, sharing personal and general health information on social media, and interacting with individuals having similar health conditions on social media (*P* > .05). Additionally, sex showed no statistically significant differences between groups (*P* = .056).

### Regression model results

The multivariable logistic regression analysis (Model 1) examining factors associated with multimorbidity status are presented in Figure 1. After adjusting for all covariates, age and income emerged as statistically significant predictors of multimorbidity. Specifically, each additional year of age was associated with significantly increased odds of having multiple chronic conditions (OR: 1.02, 95% CI: 1.01-1.03, *P* < .001). Participants in the highest income category (≥$75 000) demonstrated substantially lower odds of multimorbidity compared to those in the lowest income group (<$20 000) (OR: 0.34, 95% CI: 0.21-0.54, *P* < .001). The middle-income group ($20 000–$74 999) also showed lower odds, though to a lesser extent (OR: 0.67, 95% CI: 0.48-0.93, *P* < .05). In contrast, health insurance status, sex, race/ethnicity, and education were not significantly associated with multimorbidity in the fully adjusted model (all *P* > .05). Notably, neither the number of DHTs used nor frustration with DHTs was independently associated with multimorbidity status after adjustment for sociodemographic factors. These findings indicate that older age and lower SES, particularly income level, are the primary demographic risk factors for multimorbidity in this population.

**Figure 1.**
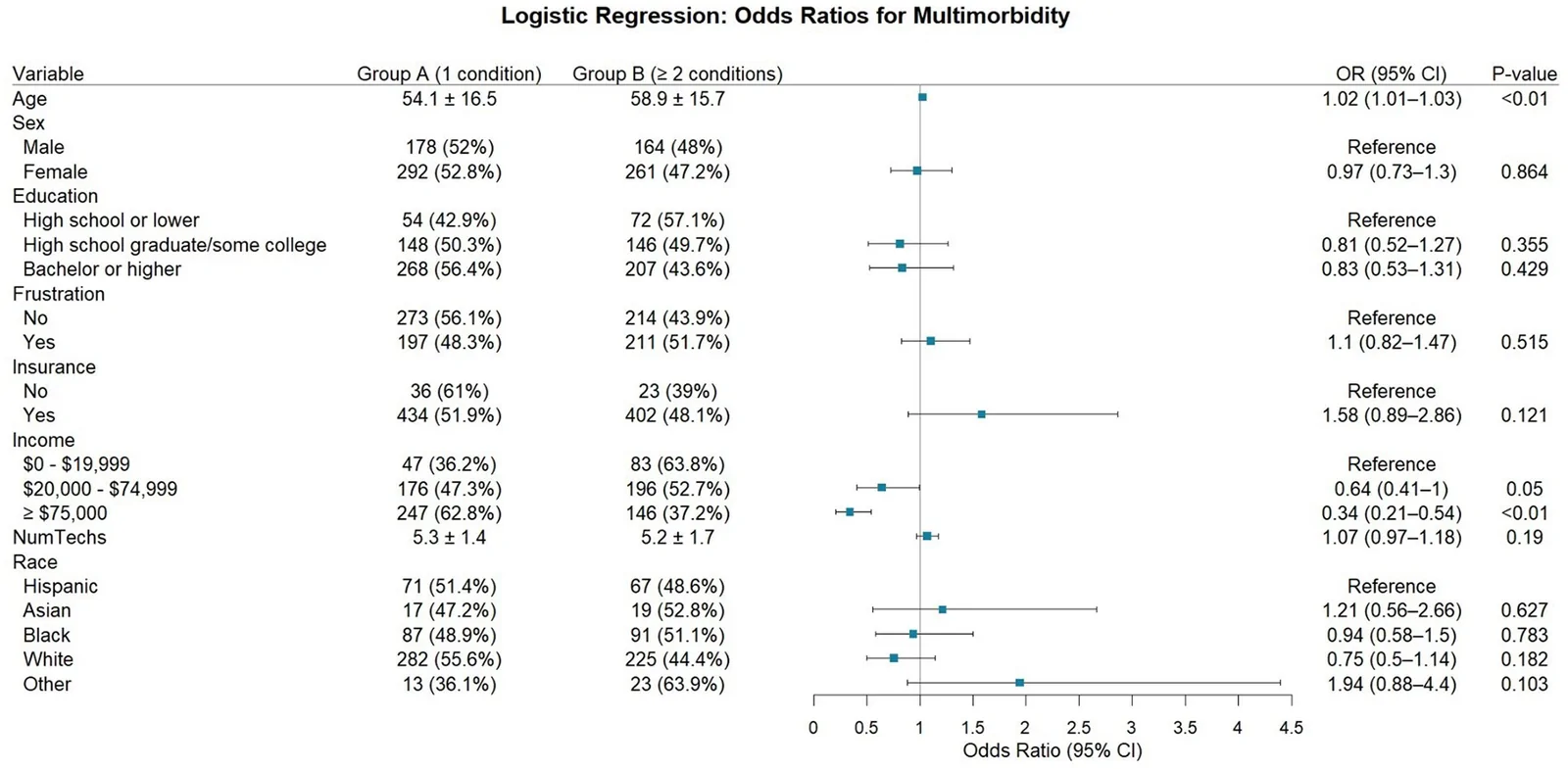
Multivariable logistic regression on predictors of multimorbidity.

The results of the multivariable logistic regression examining predictors of frustration with DHTs are shown in Figure 2. After adjustment for sociodemographic, clinical, and technology-related factors, several variables demonstrated significant associations with frustration. Older age was associated with slightly increased odds of frustration (OR: 1.02, 95% CI: 1.01-1.03, *P* < .01), with each additional year of age increasing the odds of reporting frustration by approximately 2%. Female was associated with 46% higher odds of frustration relative to male (OR: 1.46, 95% CI: 1.09-1.97, *P* = .013). Participants in the highest income group (≥$75 000) reported significantly less frustration compared to those in the lowest income category (<$20 000) (OR: 0.50, 95% CI: 0.31-0.80, *P* < .01), representing a 50% reduction in the odds. The middle-income group showed an intermediate effect (OR: 0.72, 95% CI: 0.51-1.01, *P* = .057), approaching but not reaching statistical significance. Similarly, higher education was protective, with bachelor’s degree holders showing 40% lower odds of frustration compared to those with high school education or less (OR: 0.60, 95% CI: 0.42-0.86, *P* < .01). The number of DHTs used was significantly associated with reduced frustration, with each additional technology type associated with 22% lower odds of reporting frustration (OR: 0.78, 95% CI: 0.71-0.87, *P* < .01). The presence of multimorbidity itself was not significantly associated with frustration levels after controlling for other factors (OR: 1.15, 95% CI: 0.89-1.48, *P* > .05). Additionally, no significant associations were observed for race/ethnicity or health insurance status (all *P* > .05).

**Figure 2.**
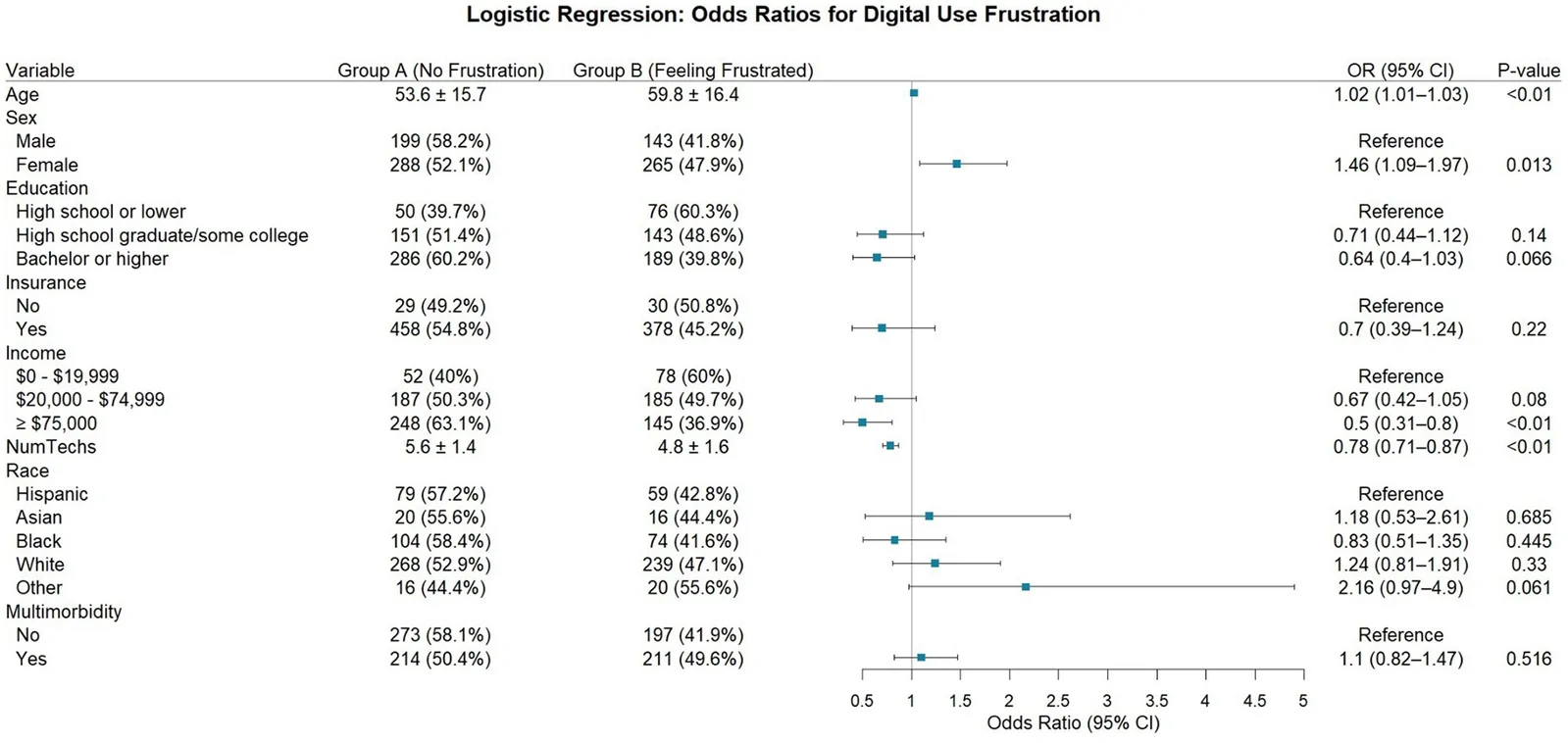
Multivariable logistic regression on predictors of DHT frustration.

### Subgroup analysis

#### Subgroup analysis by multimorbidity and DHTs adoption versatility

To further elucidate the relationship between multimorbidity, DHT usage intensity, and user frustration, participants were classified into 4 phenotypic groups based on multimorbidity status (single condition vs multiple conditions) and level of DHT adoption (<6 technologies vs ≥6 technologies). A threshold of 6 technologies was selected to define high DHT versatility based on the 75th percentile of the distribution, ensuring clinical relevance and adequate sample sizes across groups. Frustration prevalence across these groups was visualized using a 2 × 2 heatmap (Figure 3), and detailed group characteristics are presented in Table 3.

**Figure 3.**
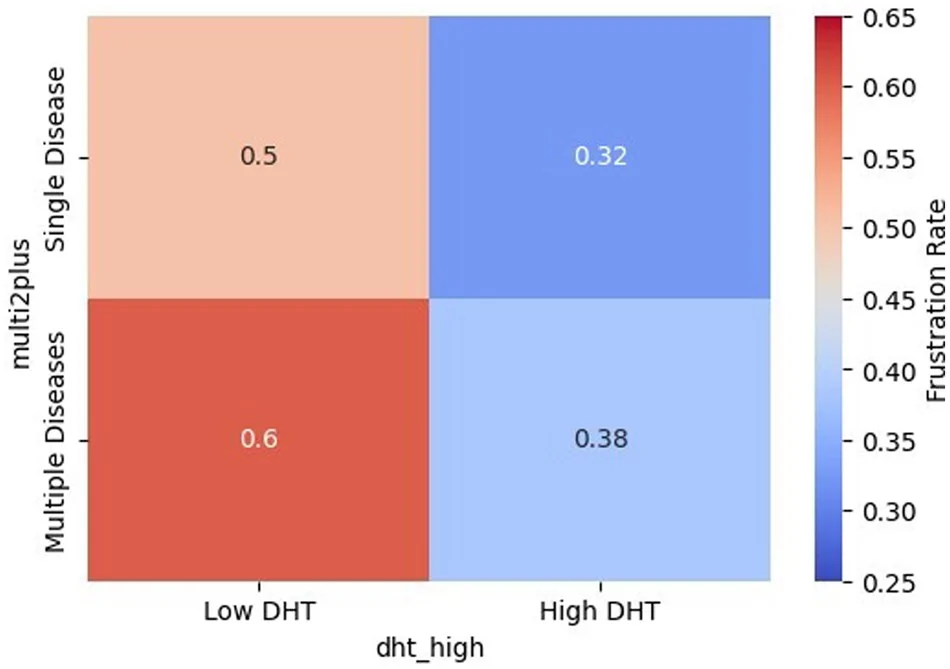
Heatmap of DHT frustration prevalence by multimorbidity status and technology usage intensity.

**Table 3.** Characteristics of phenotypic subgroups defined by multimorbidity status and DHT usage intensity.

Variable	Group 1	Group 2	Group 3	Group 4	*P*-value
	*n* = 248	*n* = 222	*n* = 219	*n* = 206	
Age	56.7 ± 16.0	51.1 ± 16.6	61.6 ± 16.3	56.1 ± 14.5	<.001
Frustrated with DHT					<.001
No	123 (49.6%)	150 (67.6%)	87 (39.7%)	127 (61.7%)	
Yes	125 (50.4%)	72 (32.4%)	132 (60.3%)	79 (38.3%)	
Income ranges					<.001
$0–$19 999	27 (10.9%)	20 (9.0%)	57 (26.0%)	26 (12.6%)	
$20 000–$74 999	110 (44.4%)	66 (29.7%)	105 (47.9%)	91 (44.2%)	
≥$75 000	111 (44.8%)	136 (61.3%)	57 (26.0%)	89 (43.2%)	
Education					<.001
High school or lower	36 (14.5%)	18 (8.1%)	54 (24.7%)	18 (8.7%)	
High school graduate/some college	83 (33.5%)	65 (29.3%)	85 (38.8%)	61 (29.6%)	
Bachelor or higher	129 (52.0%)	139 (62.6%)	80 (36.5%)	127 (61.7%)	

The 4 resulting phenotype groups were:

Group 1: Single condition, low DHT versatility (*n* = 248)
Group 2: Single condition, high DHT versatility (*n* = 222)
Group 3: Multiple conditions, low DHT versatility (*n* = 219)
Group 4: Multiple conditions, high DHT versatility (*n* = 206)

Significant differences were observed across the 4 phenotypes in age, frustration prevalence, income distribution, and education (all *P* < .001). Group 2 was the youngest (mean age 51.1 ± 16.6 years) and exhibited the lowest frustration rate (32.4%). Conversely, Group 3 was the oldest (mean age 61.6 ± 16.3 years) and reported the highest frustration rate (60.3%). Groups 1 and Group 4 demonstrated intermediate and comparable age profiles (56.7 ± 16.0 and 56.1 ± 14.5 years, respectively), though frustration was notably higher in Group 1 (50.4%) than in Group 4 (38.3%). Socioeconomic patterns further distinguish between the groups. Group 2 had the highest proportion of participants with a high income (≥$75 000: 61.3%) and bachelor’s degree or higher (62.6%). In contrast, Group 3 had the highest concentration of individuals in the lowest income bracket (<$20 000: 26.0%) and the highest proportion with high school education or less (24.7%). Groups 1 and 4 showed intermediate socioeconomic profiles; no significant differences were observed across groups in race/ethnicity, sex, or health insurance status (all *P* > .05). Individuals with multimorbidity but broad DHT adoption (Group 4) reported frustration levels with digital tools comparable to younger individuals with single conditions and similar technology versatility (Group 2). This pattern suggests that digital proficiency and successful integration of multiple technologies may mitigate the frustration specifically associated with navigating complex digital health ecosystems, though it does not necessarily reduce the underlying complexity of managing multiple chronic conditions. These “successful adopters” may have developed skills to efficiently manage multiple platforms, reducing the digital burden even as clinical complexity remains high.

#### Subgroup analysis by multimorbidity and frustration status

Participants were also stratified into 4 groups based on multimorbidity status (single vs multiple conditions) and frustration with DHTs (not frustrated vs frustrated) to examine how the interaction between diseases and technology-related frustration influences the adoption intensity of DHTs. The outcome of interest was high DHT versatility. Group differences in DHT adoption rates were visualized using the 2 × 2 heatmap (Figure 4), and detailed characteristics are summarized in Table 4.

**Figure 4.**
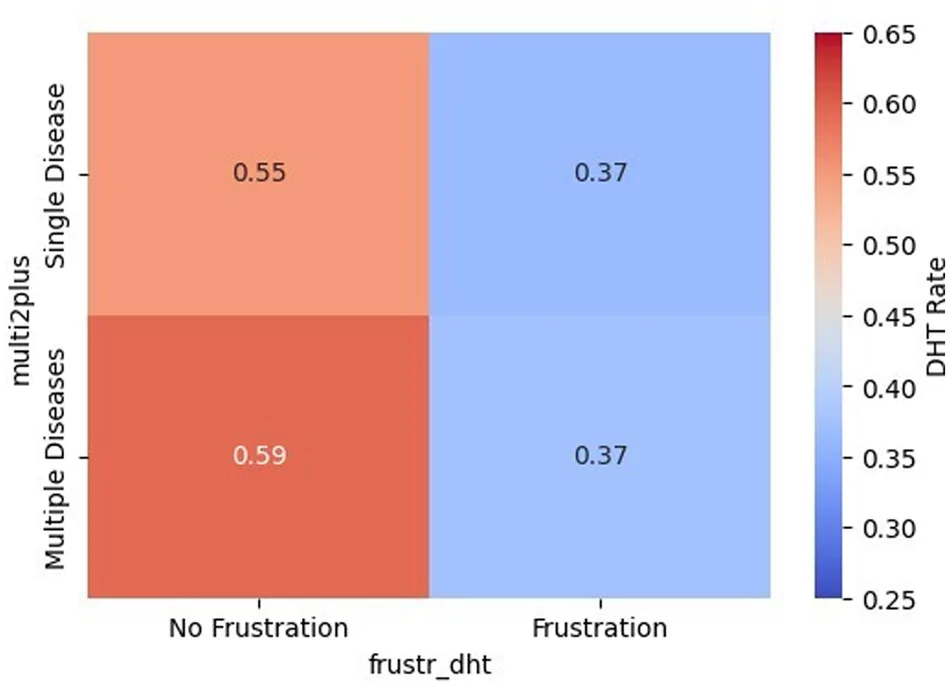
Heatmap of high DHT adoption by multimorbidity status and frustration status.

**Table 4.** Characteristics of phenotypic subgroups defined by multimorbidity status and DHT frustration.

Variable	Group A	Group B	Group C	Group D	*P*-value
	*n* = 273	*n* = 214	*n* = 211	*n* = 197	
Age	51.8 ± 16.1	57.3 ± 16.5	55.9 ± 14.8	62.1 ± 16.0	<.001
Frustrated with DHT					<.001
No	123 (45.1%)	125 (63.5%)	87 (40.7%)	132 (62.6%)	
Yes	150 (54.9%)	72 (36.5%)	127 (59.3%)	79 (37.4%)	
Income ranges					<.001
$0–$19 999	16 (5.9%)	31 (15.7%)	36 (16.8%)	47 (22.3%)	
$20 000–$74 999	98 (35.9%)	78 (39.6%)	89 (41.6%)	107 (50.7%)	
≥$75 000	159 (58.2%)	88 (44.7%)	89 (41.6%)	57 (27.0%)	
Education					.0002
High school or lower	20 (7.3%)	34 (17.3%)	30 (14.0%)	42 (19.9%)	
High school graduate/some college	85 (31.1%)	63 (32.0%)	66 (30.8%)	80 (37.9%)	
Bachelor or higher	168 (61.5%)	100 (50.8%)	118 (55.1%)	89 (42.2%)	

The 4 resulting phenotype groups were:

Group A: Single condition, not frustrated (*n* = 273)
Group B: Single condition, frustrated (*n* = 214)
Group C: Multiple conditions, not frustrated (*n* = 211)
Group D: Multiple conditions, frustrated (*n* = 197)

Significant differences were observed across the 4 groups in age, prevalence of high DHT versatility, income distribution, and education (*P* < .01 for all comparisons). Group A was the youngest (mean age 51.8 ± 16.1 years) and exhibited a high rate of intensive DHT use (55.0% using 6 or more technologies). Group C, though slightly older (mean age 55.9 ± 14.8 years), demonstrated an even higher level of technology adoption (59.3%), representing the highest DHT engagement rate among all groups. In stark contrast, Group B and Group D showed similarly low rates of high DHT versatility (36.5% and 37.4%, respectively) despite Group D being substantially older (mean age 62.1 ± 16.0 years) and having greater clinical complexity. Group A had the highest proportion of individuals with high income (58.2%) and bachelor’s degree or higher (61.5%). Group D showed the opposite pattern, with the highest concentration of low-income (22.3% earning < $20 000) and low-education (19.9% with high school or less) individuals, while Groups B and C occupied intermediate positions.

#### Digital literacy and operational competence profiles

To further characterize digital health literacy and operational competence across phenotypic subgroups, 2 radar charts were generated to visualize self-reported confidence and performance across 3 key dimensions: (1) the ability to use health-related apps without requesting assistance, (2) possessing the skills necessary to find reliable health information online, and (3) experiencing no technical problems during telehealth visits. Results are displayed in Figure 5A (grouped by multimorbidity and frustration status) and Figure 5B (grouped by multimorbidity and DHT usage level).

**Figure 5.**
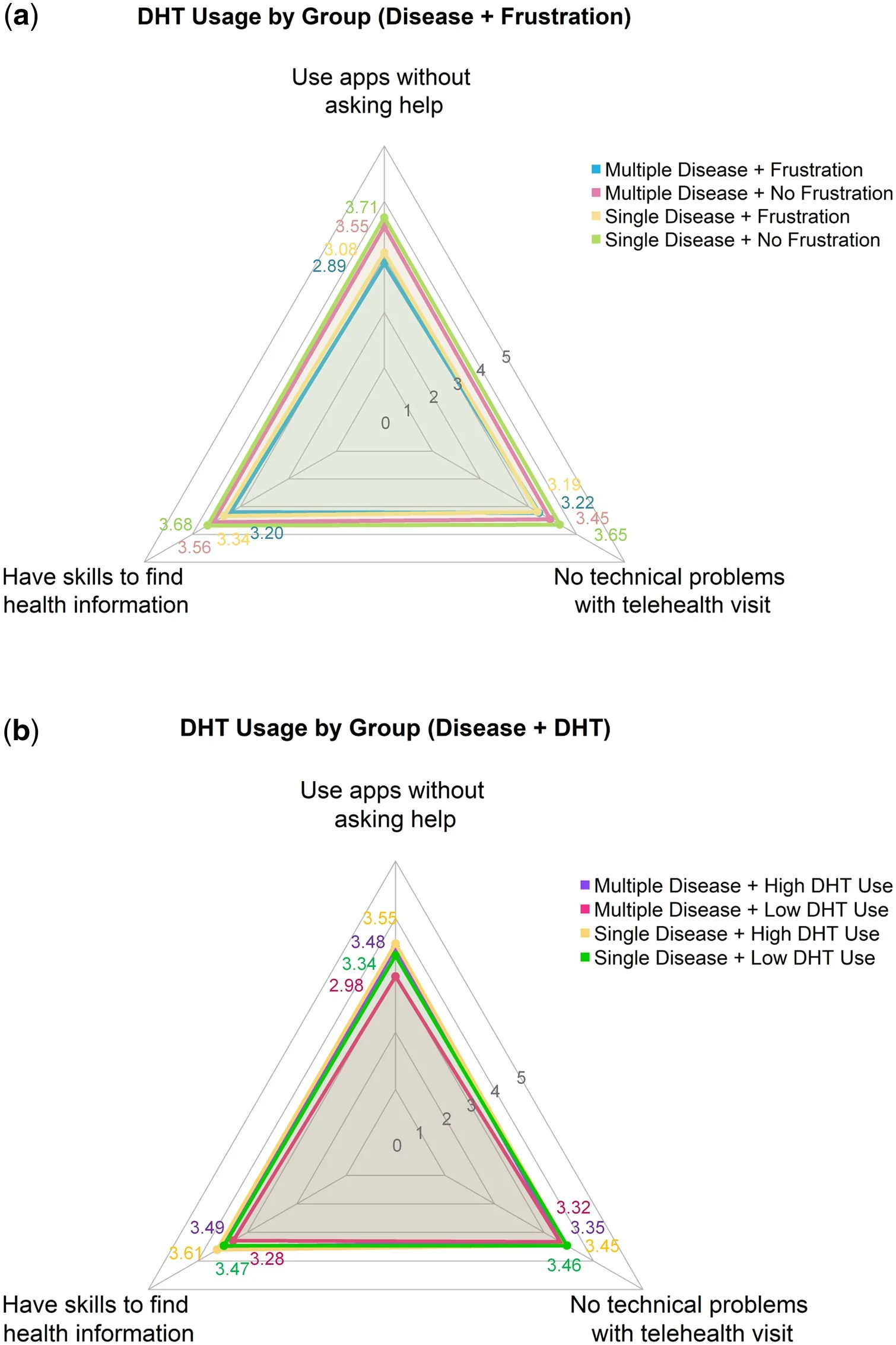
(A) Digital health literacy and operational competence profiles by multimorbidity status and frustration status. Each axis represents the percentage of participants reporting confidence or success in that domain: (1) ability to use health apps without assistance, (2) skills to find reliable health information online, and (3) experiencing no technical problems during telehealth visits. Larger triangular areas indicate more comprehensive digital competence across all 3 domains. Group A (single condition, not frustrated) demonstrates the most expansive profile, while Group D (multimorbidity, frustrated) shows the most restricted profile, indicating substantial digital capability gaps in this vulnerable subgroup. (B) Digital health literacy and operational competence profiles by multimorbidity status and digital health technology adoption versatility. The radar format reveals distinct patterns: for asynchronous tool competence (using apps, finding information), a clear gradient emerges with technology adoption level. However, for synchronous telehealth competence, multimorbidity status appears more determinative than technology versatility, with both multimorbidity groups (Groups 3 and 4) showing similar and lower levels of problem-free telehealth experiences despite their differences in overall DHT adoption. This dissociation suggests that telehealth encounters may impose unique demands not captured by general DHT proficiency.

Figure 5A reveals clear gradients in self-efficacy and technical competence when participants are grouped by diseases and frustration. The group with a single condition and no frustration exhibited the highest proficiency across all 3 domains, forming the largest radar area and indicating strong digital health capabilities. They were followed closely by the multimorbidity but not frustrated group, suggesting that the absence of frustration is strongly associated with higher perceived digital capability, even in the presence of multiple chronic conditions. The 2 frustrated groups (single condition with frustration and multimorbidity with frustration) showed relatively lower scores across all dimensions, with the multimorbidity and frustrated group demonstrating the lowest overall digital literacy profile. This pattern underscores frustration as a key marker of reduced digital competence, compounded by clinical complexity.

Figure 5B revealed a nuanced pattern. For 2 dimensions—the skills to use apps independently and to find health information online—a consistent gradient was observed: single condition with high DHT versatility > multimorbidity with high DHT versatility > single condition with low DHT versatility > multimorbidity with low DHT versatility. This gradient suggests that technology adoption level was associated with self-reported digital literacy and that these skills may be enhanced through regular technology use. However, both single condition groups (high and low DHT versatility) reported similar and higher levels of problem-free telehealth experiences, substantially outperforming both multimorbidity groups, which clustered at lower levels regardless of their DHT adoption intensity.

## Discussion

This study employed a multidimensional analytical approach to examine the complex interrelationships between multimorbidity, DHT use, and user frustration, revealing key patterns that advance our understanding of digital engagement among populations with chronic diseases. First, we quantify frustration as a patient-reported outcome measure, demonstrating that 57.6% of adults with chronic conditions experience digital burden—a prevalence that varies systematically by clinical and socioeconomic factors. Second, we reveal a paradoxical relationship: while multimorbidity increases DHT use, higher engagement independently predicts lower frustration (OR = 0.78 per additional technology), suggesting technology proficiency mitigates burden. Third, we identify Group 3 (multimorbidity + low DHT versatility) as the highest-risk phenotype with 60.3% frustration prevalence, providing a specific target for digital literacy interventions. These findings extend beyond established SES gradients to characterize how frustration manifests and is distributed across population subgroups.

Our analyses demonstrated that individuals with multiple chronic conditions report higher overall levels of frustration with DHTs despite using more types of technologies than those with single conditions. This paradox can be understood through the lens of cumulative burden. The current DHT ecosystem is predominantly characterized by condition-specific applications and platforms, which forces patients with complex care needs to navigate multiple non-integrated devices and applications to manage their health comprehensively.^9^ This fragmentation creates substantial coordination demands, increases the cognitive load required to synthesize information across platforms, and amplifies the risk of technical difficulties, duplicate data entry, and conflicting care recommendations. However, our finding that greater intensity of DHT adoption was independently associated with reduced frustration suggests that this relationship is not linear or unidirectional. The observation that individuals with multimorbidity who were highly engaged with DHTs (≥6 technologies) exhibited frustration levels comparable to their healthier counterparts with similar technology use indicates that technology proficiency and the development of integrated personal digital health ecosystems may effectively mitigate the potential burden of technology use.^18^ This pattern may reflect that individuals who successfully integrate multiple technologies into their routines develop enhanced digital literacy and self-efficacy; or alternatively, it may indicate that those with lower frustration tolerance are more likely to persist in adopting additional technologies. Longitudinal research is needed to disentangle these temporal relationships and identify the mechanisms through which technology proficiency develops.

Our findings suggest several evidence-based intervention strategies, though their effectiveness requires empirical validation. The association between higher DHT use and lower frustration (OR = 0.78) suggests that enhancing digital proficiency may reduce burden, supporting competency-based training programs tailored to baseline skill levels. The Group 3 phenotype (older, lower SES, high frustration) indicates need for multi-level support: simplified interfaces reducing cognitive load, peer mentorship connecting successful adopters (Groups 2 and 4) with struggling users, and accessible technical assistance. The persistent SES gradients (income OR = 0.50, education OR = 0.60 for highest vs lowest categories) underscore that proficiency interventions alone are insufficient—structural barriers including device access, connectivity, and system-level interoperability should be addressed simultaneously. However, our cross-sectional design cannot establish whether frustration reduction follows from increased proficiency or whether lower baseline frustration enables adoption. Longitudinal intervention studies are essential to test these mechanistic pathways and evaluate implementation strategies.^19^

A further central finding of this study is the substantial moderating influence of SES on digital health engagement outcomes. Income and education emerged as foundational determinants of both technology adoption patterns and user experience quality.^20^ Individuals with higher SES were significantly more likely to adopt multiple DHTs and reported substantially lower frustration levels, irrespective of their multimorbidity. This persistent socioeconomic gradient, which was evident across multiple analytical approaches and remained robust in fully adjusted models, suggests that socioeconomic barriers may represent more fundamental determinants of digital health engagement than clinical factors alone.^21^ The mechanisms underlying this socioeconomic gradient are likely multifactorial and include differential access to high-quality devices and reliable internet connectivity, variations in baseline digital literacy and technical skills, differences in the availability of informal support networks for troubleshooting technical problems, and potential disparities in the quality and usability of DHTs provided through different healthcare settings or insurance plans. Consequently, unless DHTs are intentionally designed with equity as a foundational principle, they risk widening pre-existing health disparities by creating additional structural barriers for socioeconomically marginalized populations.^20^ This finding has critical implications for policy and implementation: digital health initiatives should incorporate explicit equity metrics, provide robust technical support infrastructure, and ensure that DHT design and deployment strategies actively address rather than perpetuate socioeconomic disparities.

The interaction between multimorbidity and DHT use revealed distinct digital engagement phenotypes with important practical implications for targeted interventions. We identified a particularly informative phenotype comprising individuals with multimorbidity and high DHT versatility (Group 4), whose frustration levels were comparable to those of individuals with lower disease burden and similar technology use patterns (Group 2). This pattern suggests that technology proficiency and the successful integration of multiple DHTs into cohesive personal digital health ecosystems may effectively mitigate the digital burden typically associated with complex care needs. These “successful adopters” may benefit from characteristics such as higher baseline digital literacy, stronger self-efficacy, access to better technical support, or exposure to more user-friendly and interoperable systems.^22^ Conversely, we identified a high-risk phenotype characterized by older adults with multimorbidity, lower SES, and limited technology adoption (Group 3). This profile demonstrated the highest frustration levels (60.3%) and aligns with documented barriers such as limited digital literacy, physical limitations (eg, visual impairment, reduced manual dexterity), cognitive challenges (eg, reduced working memory, slower processing speed), and practical difficulties with fundamental operations such as connecting to the internet, troubleshooting technical problems, or navigating complex interfaces.^23^ These individuals are at highest risk for digital exclusion—a phenomenon where marginalized communities with lower digital literacy and internet access are further disadvantaged by technology-dependent healthcare delivery models.^24^ This phenotypic stratification highlights how technology adoption and proficiency may empower some patients with complex conditions, while simultaneously underscoring the urgent need for targeted interventions to support vulnerable subgroups as healthcare systems become increasingly digitalized.^25^

The radar chart analyses provided insights into distinct dimensions of digital literacy and operational competence. While overall frustration with DHTs primarily related to general digital self-efficacy and confidence in using asynchronous tools (eg, health apps and online information seeking), multimorbidity status specifically predicted technical difficulties during synchronous telehealth visits.^26^ This dissociation may suggest several possibilities. First, frustration with DHTs appears to stem more from practical operational challenges than from inherent technology complexity per se. These operational challenges include navigating non-intuitive interfaces, managing interoperability issues between disparate systems, coordinating information across multiple platforms, and coping with inadequate training tailored to individual needs and limitations.^27^ Second, the finding that multimorbidity predicts telehealth technical difficulties independently of overall DHT adoption suggests that synchronous video-based encounters may impose unique cognitive, physical, or technical demands that are particularly challenging for individuals managing multiple conditions.

These demands may include the need to manage medication schedules and symptoms during appointments,^4^ coordinate care across multiple specialists simultaneously,^9^ communicate complex medical histories under time pressure,^28^ or troubleshoot audio-visual problems in real-time while experiencing health-related stress.^29^ Building upon prior research,^20^ these findings highlight a critical need for a dual-pronged approach: (1) development of simplified, more intuitive user interfaces that reduce cognitive load^27^ and accommodate physical limitations,^23^ employing principles of universal design,^30^ and (2) implementation of personalized, competency-based training programs^31^ that address specific skill gaps and the physical or cognitive limitations^24^ common among patients with high multimorbidity burden. Additionally, telehealth platforms may require specialized optimization, such as pre-call technical checks, simplified connection protocols, asynchronous pre-visit information gathering, and dedicated technical support hotlines, to better accommodate patients with complex chronic conditions.^29^ However, the HINTS telehealth question assesses only whether problems occurred, not their nature, frequency, or impact. We cannot determine whether reported difficulties reflect patient-side factors (internet bandwidth, device quality), provider-side factors (platform usability), or interaction effects.

The non-significance of race/ethnicity, sex, and health insurance status in the fully adjusted frustration models, while age, income, and education remained significant, suggests that digital divides may be evolving from primarily access-based disparities toward proficiency-based disparities. As digital technologies become more ubiquitous and internet connectivity improves, the differential ability of individuals to develop and effectively apply digital skills is emerging as the primary driver of digital health inequality.^32^ This transition is consistent with scholarship on the “second-level digital divide,” which posits that disparities in how people use technology (skills, self-efficacy, beneficial use patterns) may be more consequential than disparities in whether they have access to technology.^33^ However, this interpretation requires several important caveats. First, HINTS' digital administration may systematically exclude populations with severe access barriers, masking true access-based disparities. Second, persistent SES gradients indicate structural inequities continue shaping engagement through mechanisms encompassing both access (device quality, connectivity) and proficiency (skills, self-efficacy).^34^ Third, our broad frustration measure cannot distinguish whether reported difficulties reflect fundamental access issues vs operational challenges among users with adequate access. While scholarship on “second-level digital divides” emphasizes skills over access, our data cannot definitively establish this transition—both access and proficiency barriers likely coexist and require simultaneous intervention.^35^

These findings underscore that while improving digital literacy and skills training is essential, these efforts should be complemented by continued attention to structural barriers (affordability, connectivity infrastructure, device quality) and by fundamental improvements in DHT design that reduce the baseline skill requirements for effective use.^36^ The goal should not be merely to train more patients to use complex systems, but to create simpler, more intuitive systems that accommodate diverse user needs and capabilities from the outset. These findings also highlight the need for multi-level interventions. At the system level, healthcare organizations and policymakers should prioritize the development of interoperable platforms that reduce the burden of managing multiple applications,^37^ establish universal design principles that accommodate diverse user capabilities,^38^ and implement equity metrics to monitor and address digital health disparities.^39^ At the individual level, targeted interventions should focus on personalized digital literacy training, peer support programs, and accessible technical assistance,^18^ particularly for older adults with multimorbidity and low SES who are at highest risk for digital exclusion.^40^^,^^41^ Intervention studies are needed to test strategies for reducing frustration and improving digital health equity.^31^ Promising approaches include personalized digital literacy training programs tailored to individual skill levels and learning styles,^30^ peer support and mentorship programs connecting successful adopters with those struggling with technology,^31^ technical support hotlines staffed by specialists familiar with the unique needs of older adults and those with chronic conditions,^18^ and co-design processes that involve patients with multimorbidity in the development and iterative refinement of DHTs to ensure they meet real-world needs.^42^

### Limitations

This study has several limitations. First, the cross-sectional design cannot provide causal inference regarding the observed relationships. Self-reported technology use may be subject to recall bias and social desirability bias. While our phenotypes identify vulnerable subgroups, they do not reveal the mechanisms underlying frustration or provide direct evidence for intervention effectiveness. The proposed solutions (digital literacy training, technical support, universal design) arise from general digital equity principles rather than from mediator analyses within our data. Future research should employ mediation analysis to identify modifiable factors (eg, self-efficacy, social support, device quality) that explain observed associations, and conduct intervention trials testing whether addressing these factors reduces frustration and improves outcomes. Second, the DHT threshold (ie, 6 technologies), while statistically derived, its clinical relevance requires practical validation in diverse populations. Third, the DHT threshold (≥6 technologies), while statistically derived from the 75th percentile, represents a pragmatic rather than theoretically validated cutoff. The clinical meaningfulness of this distinction requires validation through objective usage metrics (eg, frequency, duration, feature utilization) and patient-reported outcomes. Future studies should employ data-driven approaches such as latent class analysis or clustering algorithms to identify naturally occurring subgroups based on multidimensional usage patterns rather than single cutpoints. Finally, as the HINTS survey is administered primarily through digital platforms, it may systematically underrepresent populations with limited digital access or literacy, potentially underestimating the true digital divide and the frustration burden among the most vulnerable groups. Since the survey was conducted exclusively in the United States, the findings’ generalizability may be limited by the cultural and healthcare system context, requiring validation in other national settings. Future research could employ longitudinal designs to track how technology adoption patterns evolve with changing health status, and incorporate objective measures of digital engagement, such as platform usage metrics and device-collected data, to complement self-reported measures.

## Conclusion

This study provides empirical evidence that digital health engagement is not merely a function of clinical need or technology availability, but rather reflects complex interplays between socioeconomic resources, technology proficiency, and clinical complexity. The identified phenotypes may offer a novel framework for identifying vulnerable subgroups that would benefit most from tailored digital health strategies and for understanding the mechanisms through which technology may empower some patients while excluding others. The goal should be to create digital health ecosystems that genuinely reduce rather than merely redistribute the burden of chronic disease management. This requires moving beyond a narrow focus on technology adoption rates to embrace a more comprehensive vision of digital health equity that attends to user experience, operational competence, and the structural determinants of meaningful engagement. Only through such patient-centered approaches can DHT fulfill their potential to support improved outcomes and reduced burden for all patients with multimorbidity.

## Data Availability

Data from the Health Information National Trends Survey (HINTS 7) are publicly available from the National Cancer Institute at https://hints.cancer.gov/
